# The human microbial exposome: expanding the Exposome-Explorer database with gut microbial metabolites

**DOI:** 10.1038/s41598-022-26366-w

**Published:** 2023-02-02

**Authors:** Vanessa Neveu, Geneviève Nicolas, Adam Amara, Reza M. Salek, Augustin Scalbert

**Affiliations:** Nutrition and Metabolism Branch, International Agency for Research on Cancer (IARC), 25 avenue Tony Garnier, CS 90627, 69366 Lyon Cedex 07, France

**Keywords:** Computational biology and bioinformatics, Microbiology, Biomarkers, Risk factors

## Abstract

Metabolites produced by the gut microbiota play an important role in the cross-talk with the human host. Many microbial metabolites are biologically active and can pass the gut barrier and make it into the systemic circulation, where they form the gut microbial exposome, i.e. the totality of gut microbial metabolites in body fluids or tissues of the host. A major difficulty faced when studying the microbial exposome and its role in health and diseases is to differentiate metabolites solely or partially derived from microbial metabolism from those produced by the host or coming from the diet. Our objective was to collect data from the scientific literature and build a database on gut microbial metabolites and on evidence of their microbial origin. Three types of evidence on the microbial origin of the gut microbial exposome were defined: (1) metabolites are produced in vitro by human faecal bacteria; (2) metabolites show reduced concentrations in humans or experimental animals upon treatment with antibiotics; (3) metabolites show reduced concentrations in germ-free animals when compared with conventional animals. Data was manually collected from peer-reviewed publications and inserted in the Exposome-Explorer database. Furthermore, to explore the chemical space of the microbial exposome and predict metabolites uniquely formed by the microbiota, genome-scale metabolic models (GSMMs) of gut bacterial strains and humans were compared. A total of 1848 records on one or more types of evidence on the gut microbial origin of 457 metabolites was collected in Exposome-Explorer. Data on their known precursors and concentrations in human blood, urine and faeces was also collected. About 66% of the predicted gut microbial metabolites (*n* = 1543) were found to be unique microbial metabolites not found in the human GSMM, neither in the list of 457 metabolites curated in Exposome-Explorer, and can be targets for new experimental studies. This new data on the gut microbial exposome, freely available in Exposome-Explorer (http://exposome-explorer.iarc.fr/), will help researchers to identify poorly studied microbial metabolites to be considered in future studies on the gut microbiota, and study their functionalities and role in health and diseases.

## Introduction

The gut microbiota plays an important role in human health and modulates risk of various diseases such as obesity, cardiovascular diseases, diabetes, colorectal cancer, inflammatory bowel disease, and depression^1–4^. Mechanisms are not fully elucidated but metabolites produced by the gut microbiota play an important role in the cross-talk between the microbiota and the host^3,5^. Many of these gut microbial compounds have shown some biological activities. For example, secondary bile acids have anti-inflammatory properties and may limit risk of inflammatory bowel disease^6^. Short chain fatty acids provide energy to the gut mucosa, improve glucose homeostasis and prevent metabolic disorders in rodents and humans^3^. Branched chain amino acids induce insulin resistance and have been associated with obesity and diabetes^7^. Equol, a biotransformation product of the soy phytoestrogen daidzein, shows an estrogenic potency higher than that of its parent compound^8^.

Gut microbial metabolites exert local effects on the gut mucosa or, after absorption through the intestinal barrier, distal effects on inner tissues. Many of them are found in the systemic circulation and in inner tissues^9^. Importantly, gut microbial metabolites can also be seen as a read out of microbiota functionalities and dysbiosis^2^. Their study in blood may help deciphering the role of the gut microbiota in health and diseases, more particularly in large cohort studies where no faecal samples have been collected.

Microbial metabolites are small molecules (*M* < 1,000 Da) either synthesized de novo by the bacteria, or formed by bacterial biotransformation of xenobiotics (dietary compounds, drugs) or host-derived compounds^10,11^. A large diversity of compounds is known, belonging to various chemical classes such as short chain fatty acids, bile acids, choline metabolites, phenols, indole derivatives, vitamins, polyamines and lipids^5^. The gut microbiota varies between individuals and is largely conserved along lifetime^12,13^. As such it can be considered as an exposure just as lifestyle, diet or pollutants, and microbial metabolites in blood or tissues can be seen as exogenous compounds, similarly to dietary compounds derived from the digestion of foods and to pollutants. We propose here to name the totality of microbial metabolites in human biospecimens, the microbial exposome. As such, the microbial exposome adds to dietary compounds, pollutants, drugs and endogenous compounds, all parts of our internal exposome, defined as the sum of all chemicals and metabolites in blood and tissues constituting our internal chemical environment^14–16^.

A major difficulty faced when studying the microbial exposome is to differentiate metabolites that are formed by the microbiota from those that are formed in human tissues or directly derived from the diet. These difficulties arise from several reasons. Firstly, gut bacteria, humans and food species share some common metabolic pathways, and metabolites in these pathways found in blood or tissues may originate from microbial activity, human tissular activity or the diet. Secondly, metabolites may have a mixed origin. They may be formed by the transformation of human or dietary precursors, and microbial metabolites may be further metabolized in the liver and other human tissues. Identifying microbial metabolites largely or exclusively formed by the microbiota is needed to get a better insight on the microbiota metabolic function in the complex environment of the host^3,10^.

The purpose of the present work was to develop a comprehensive database on gut microbial metabolites with evidence supporting their microbial origin. Data for 457 gut microbial metabolites was extracted from peer-reviewed publications, and has been curated in the Exposome-Explorer database where it can be easily searched^17^. To further enlarge coverage of gut microbial metabolites, we also compared genome-scale metabolic models (GSMMs) of gut bacterial strains to those of humans to identify metabolites uniquely formed by the microbiota. This new resource should help researchers to identify gut microbial metabolites to be considered in future studies on the gut microbiota, its functionalities and role in health and diseases.

## Results

## Collection of experimental evidence on the microbial origin of metabolites

Three types of experimental evidence were used to support the microbial origin of metabolites:

- (i) Microbial metabolites are produced by human faecal bacteria grown in vitro.
- (ii) Concentrations of microbial metabolites are reduced upon antibiotic treatment in humans or experimental animals^18^.
- (iii) Concentrations of microbial metabolites are lower in germ-free animals when compared with conventional animals^9^. Concentrations are increased by transplantation of faecal samples or gut bacterial strains to germ-free animals^19,20^.

**Figure 1:**
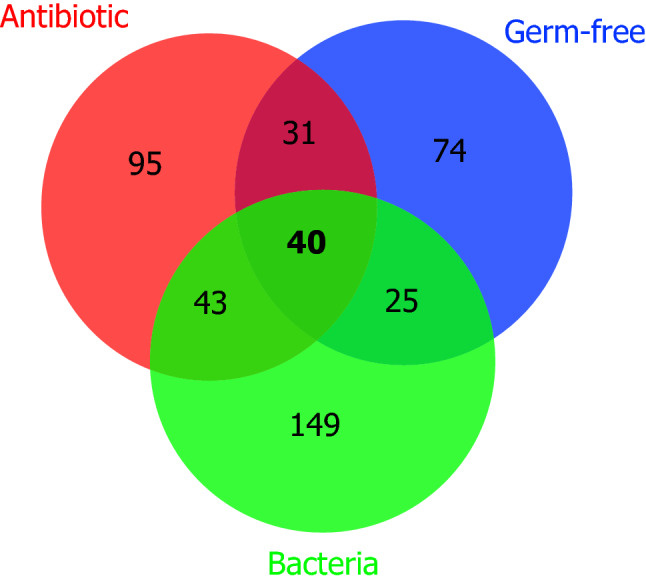
Venn diagram showing numbers of metabolites with microbial origin supported by one, two or three types of evidence. The three types of evidence are: produced by human faecal bacteria, reduced by antibiotic treatment, and reduced in germ-free animals.

**Figure 2:**
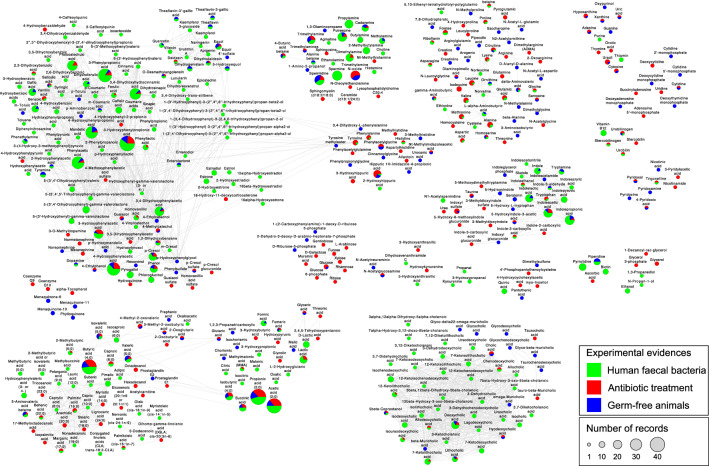
Chemical similarity network of 457 microbial metabolites in the Exposome-Explorer database. Node size is proportional to the number of records supporting their microbial origin. Colours indicate the types of experimental evidence on microbial origin: produced by human faecal bacteria, reduced by antibiotic treatment, and reduced in germ-free animals.

A total of 165 publications supporting the microbial origin of human metabolites was identified, corresponding to a total of 1848 records on evidence of microbial origin (http://exposome-explorer.iarc.fr/microbial_metabolite_identifications) for 457 metabolites (http://exposome-explorer.iarc.fr/microbial_metabolites). Most records were related to the production of these metabolites by human faecal bacteria (*n* = 1182), followed by reduction of their concentrations upon antibiotic treatment (*n* = 418) and reduction of their concentrations in germ-free animals (*n* = 248). Out of the 457 metabolites, 318 have a microbial origin supported by only one type of experimental evidence, 99 by two types, and 40 by the three types (Figs. 1 and 2). Based on their chemical structure, the 457 microbial metabolites were automatically categorized into 189 chemical classes using the ChemOnt chemical taxonomy. To facilitate our analysis, these numerous classes were manually grouped into 33 upper-level classes with more meaningful biochemical names of the ChemOnt taxonomy (Table 1, Additional file 1: Figure S1).

**Table 1:** Main classes of microbial metabolites in the Exposome-Explorer database and type of evidence supporting their microbial origin.

Metabolite class	Number of metabolites				Examples of metabolites^1^
	Total	Produced by human faecal bacteria	Reduced by antibiotic treatment	Reduced in germ-free animals	
Amino acids, peptides, and analogues	62	25	36	28	Phenylacetylglycine; Alanine; Tyrosine
Phenylpropanoids and polyketides	46	44	11	16	3-Hydroxyphenylpropionic acid; 3-Phenylpropionic acid; 4-Hydroxyphenylpropionic acid
Fatty acyls	45	30	25	6	Butyric acid (4:0); Valeric acid (5:0); Isovaleric acid (5:0)
Bile acids, alcohols and derivatives	45	33	11	12	Deoxycholic acid; 7-Ketolithocholic acid; Isochenodeoxycholic acid
Phenols	31	18	15	15	4-Hydroxyphenylacetic acid; 3,4-Dihydroxyphenylacetic acid; 3-Hydroxyphenylacetic acid
Indoles and derivatives	23	13	10	12	Indolepropionic acid; Indoleacetic acid; Indolelactic acid
Carbohydrates and carbohydrate conjugates	22	0	16	9	Glucose; *N*-Acetylglucosamine; Glycerol
Organic nitrogen compounds	22	15	10	10	Spermidine; Spermine; Trimethylamine *N*-oxide
Benzoic acids and derivatives	21	16	7	7	Protocatechuic acid; Hippuric acid; Gallic acid
Carboxylic acids and derivatives	14	11	7	9	Propionic acid (3:0); Acetic acid (2:0); Isobutyric acid (4:0)
Benzene and substituted derivatives	12	10	3	4	Phenylacetic acid; Tyramine; Phenethylamine
Hydroxy acids and derivatives	10	4	5	2	Lactic acid; *D*-Lactic acid; 3-Hydroxypropionic acid
Steroids and steroid derivatives	10	9	2	1	Estradiol; Estrone; 5beta-Coprostanol
Alcohols and polyols	9	6	2	1	Quinic acid; Ethanol; Pantothenic acid
Nucleosides, nucleotides, and analogues	9	0	5	5	Cytidine; Deoxycytidine; Adenosine 5'-monophosphate
Organic acids and derivatives	8	1	6	4	Indoxyl sulfate; *N1*-Acetylspermidine; Taurine
Pyridines and derivatives	7	0	4	5	4-Pyridoxic acid; Pyridoxal; Nicotinic acid
Prenol lipids	7	0	3	4	Menaquinone-6; Menaquinone-10; Menaquinone-11
Keto acids and derivatives	7	1	5	4	4-Methyl-2-oxovaleric acid; 2-Oxoglutaric acid; 3-Methyl-2-oxobutyric acid
Purines and purine derivatives	7	1	4	6	Hypoxanthine; Adenine; Xanthine
Carbonyl compounds	6	4	2	0	3-Hydroxypropanal; 4-Hydroxybenzaldehyde; 3,4-Dihydroxybenzaldehyde
Lipids and lipid-like molecules	6	1	5	0	Isocholic acid; Sphingomyelin (d18:2/18:0); 1-Decanoyl-rac-glycerol
Tetrapyrroles and derivatives	5	4	4	1	Vitamin B12; Urobilinogen; Urobilin
Pyrimidines and pyrimidine derivatives	5	1	4	2	Uracil; Thiamin; Cytosine
Organoheterocyclic compounds	4	3	1	1	Pyrrolidine; Piperidine; Biotin
Imidazoles	4	0	2	3	Nt-Methylimidazoleacetic acid; Allantoin; Urocanic acid
Pteridines and derivatives	3	2	2	1	Folates; Riboflavin; 7,8-Dihydropteroic acid
Lignans, neolignans and related compounds	2	2	0	1	Enterolactone; Enterodiol
Alkaloids and derivatives	1	1	1	0	Trigonelline
Organosulfur compounds	1	0	0	1	Dimethylsulfone
Hybrid peptides	1	0	1	0	4'-Phosphopantothenoylcysteine
Phenol ethers	1	1	0	0	5-(3'-Methoxyphenyl)valeric acid
Phenol esters	1	1	0	0	2'',3''-Dihydroxyphenoxyl-3-(3',4'-dihydroxyphenyl)propionic acid
Total	457	257	209	170	

^1^The first three metabolites with larger number of records on experimental evidence.

Many of these microbial metabolites are also known as human metabolites and the relative contributions of the gut microbiota and human tissues to their formation is most often unknown. However, reduction of their concentrations upon treatment with antibiotics or in germ-free animals supports a significant contribution of the microbiota to their production. One hundred and eight metabolites from 17 different classes are produced by human faecal bacteria and also show reduction of their concentrations upon treatment with antibiotics and/or in germ-free animals (Fig. 1, Table 2).

**Table 2:** Metabolites showing at least two types of evidence supporting their microbial origin [(produced by human faecal bacteria) and (reduced upon either treatment with antibiotics OR reduced in germ-free animals)].

Metabolite class	Number of metabolites	Metabolites
Amino acids, peptides, and analogues	20	Alanine; Asparagine; Aspartic acid; Citrulline; delta-Aminovaleric acid; gamma-Aminobutyric acid; Glutamic acid; Glycine; Histidine; Homoserine; *L*-alpha-Aminobutyric acid; Leucine; Lysine; Methionine; Ornithine; Phenylalanine; Proline betaine; Proline; Tyrosine; Valine
Phenylpropanoids and polyketides	18	3-Hydroxyphenylpropionic acid; 3-Phenylpropionic acid; 3,4-Dihydroxyhydrocinnamic acid; 4-Hydroxyphenylpropionic acid; 5-Hydroxyequol*; Daidzein; Dihydrodaidzein; Dihydroferulic acid; Dihydrogenistein; Equol; Ferulic acid; m-Coumaric acid; O-Desmethylangolensin; Phenyllactic acid; Quercetin; Theaflavin*; Theaflavin-3-gallic acid*; Theaflavin-3’-gallic acid*
Fatty Acyls	11	2-Methylbutyric acid (5:0); 4-Butyric acid betaine; 5-Aminovaleric acid betaine; Behenic acid (22:0); Butyric acid (4:0); Caproic acid (6:0); Isovaleric acid (5:0); Margaric acid (17:0); Palmitic acid (16:0); Palmitoleic acid (cis-16:1n-7); Valeric acid (5:0)
Bile acids, alcohols and derivatives	7	12-Ketolithocholic acid; 7-Ketolithocholic acid; Allodeoxycholic acid; Chenodeoxycholic acid; Deoxycholic acid; Isoursodeoxycholic acid; Lithocholic acid
Phenols	8	1,2-Dihydroxybenzene; 3-Hydroxyphenylacetic acid; 3,4-Dihydroxyphenylacetic acid; 4-Hydroxyphenylacetic acid*; Dopamine; Homovanillic acid; *p*-Cresol; Phenol
Indoles and derivatives	9	Indole-3-aldehyde; Indole-3-carboxylic acid; Indole; Indoleacetic acid; Indoleethanol; Indolelactic acid; Indolepropionic acid; Tryptamine; Tryptophan
Organic nitrogen compounds	7	Alanine betaine*; Cadaverine; Dimethylamine; Methylamine; Putrescine; Spermidine; Trimethylamine
Benzoic acids and derivatives	7	2,3-Dihydroxybenzoic acid; Benzoic acid; Gallic acid; Hippuric acid; Protocatechuic acid; Salicylic acid; Vanillic acid
Carboxylic acids and derivatives	7	Acetic acid (2:0); Formic acid; Fumaric acid; Isobutyric acid (4:0); Methylmalonic acid (MMA); Propionic acid (3:0); Succinic acid
Benzene and substituted derivatives	4	2-Hydroxyphenylacetic acid; Phenethylamine; Phenylacetic acid; Tyramine
Hydroxy acids and derivatives	1	Lactic acid
Steroids and steroid derivatives	1	5beta-Coprostanol
Tetrapyrroles and derivatives	3	Stercobilinogen*; Urobilin*; Urobilinogen
Organoheterocyclic compounds	1	Piperidine
Pteridines and derivatives	2	Folates; Riboflavin
Lignans, neolignans and related compounds	1	Enterolactone
Alkaloids and derivatives	1	Trigonelline

* Concentrations in human biospecimens (blood, urine or faecal samples) are available in Exposome-Explorer for all metabolites except those marked with an asterisk.

**Figure 3:**
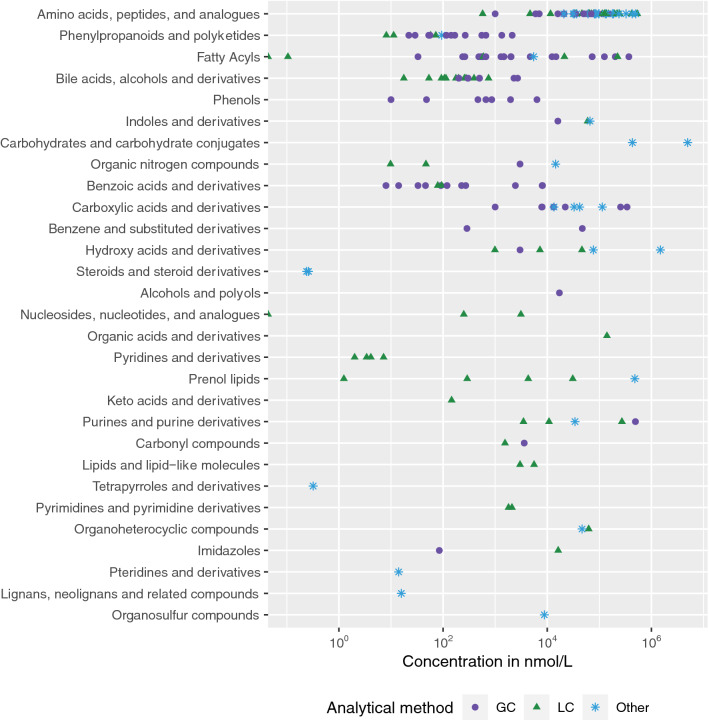
Concentrations of 152 microbial metabolites in the Exposome-Explorer database measured in human blood with different types of analytical methods. GC—gas chromatography; LC—liquid chromatography.

Information on biospecimens and analytical methods used to measure microbial metabolites in humans has been collected from the scientific literature and is presented in the ‘Concentrations’ page of the Exposome-Explorer database (http://exposome-explorer.iarc.fr/concentrations). Visualizations for this data are provided in Additional file 1: Figure S2. A large fraction of microbial metabolites (64%) shows at least one concentration value in human biospecimens, with blood and urine matrices being most widely documented. Aggregated concentrations in blood, summarized as median, vary from trace amounts to concentrations as high as 4.9 × 10^6^ nmol/L for glucose (Fig. 3).

In vitro experiments, human intervention studies and studies using experimental animals often used specific substrates biotransformed into microbial metabolites in the gut. Names of ‘substrates’ were collected in the database. These substrates added to the growth medium of faecal bacteria or administered to the subjects or experimental animals can be pure compounds, foods or food extracts. These substrates are or contain compounds assumed to be precursors of the microbial metabolites formed in the experiment. A total of 153 compounds were identified as precursors of 227 microbial metabolites. A same metabolite can be formed from a diversity of precursors, like propionic acid formed from 20 different precursors (amino acids, sugars, polysaccharides or proteins). Conversely, various microbial metabolites can be formed from one precursor. For example, 19 secondary bile acids were formed from cholic acid by various faecal bacteria grown in vitro.

## In silico predicted microbial metabolites

**Figure 4:**
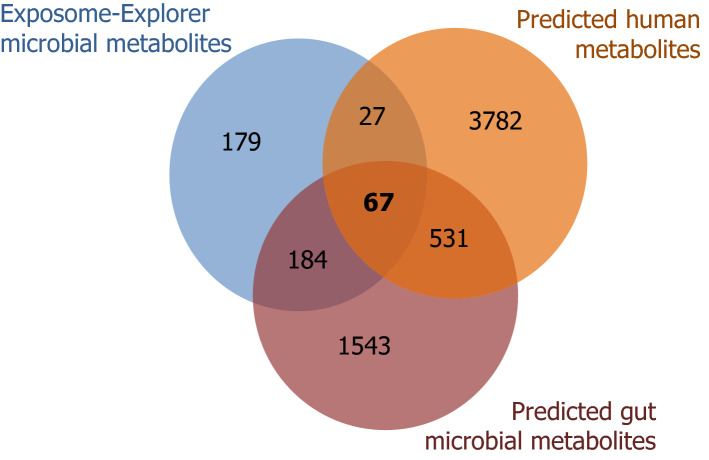
Venn diagram of gut microbial metabolites from the Exposome-Explorer database, the gut microbiota genome-scale metabolic models and the human genome-scale metabolic model.

To further explore the chemical space of the microbial exposome, GSMMs of known gut bacteria were used to compare in silico predicted metabolites with microbial metabolites curated in the Exposome-Explorer database. GSMMs are generated by using the genome information of known gut microbes and the enzymatic reactions inferred from the genome sequences. Microbial enzymatic reactions were used to predict the gut microbial exposome. A total of 2325 metabolites were predicted based on both the AGORA^21^ and MAMBO^22^ gut microbiome GSMM reconstructions. More than half (251 metabolites) of the Exposome-Explorer metabolites were found in the gut microbiome GSMMs (Fig. 4). The extent of overlap between predicted metabolites and metabolites curated in the Exposome-Explorer database varied according to chemical classes, with some classes such as polyketides, terpenoids and flavonoids being largely absent in the Exposome-Explorer database. In contrast, most of the predicted metabolites from the bile acid and amino acid classes are found in the Exposome-Explorer database.

**Figure 5:**
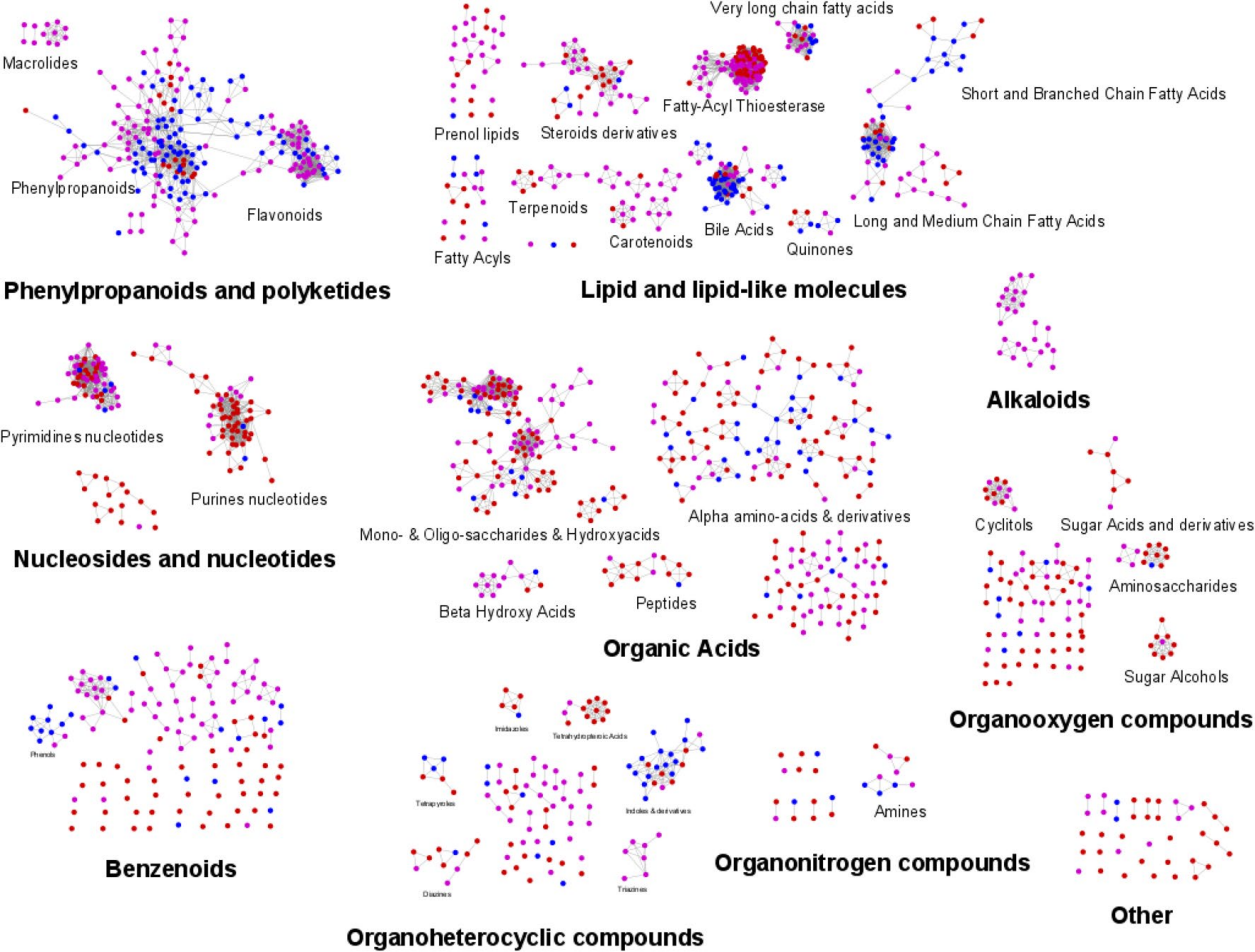
Chemical similarity networks of the 2325 predicted gut microbial metabolites and 457 microbial metabolites in the Exposome-Explorer database. Red nodes from MAMBO genome-scale metabolic model; pink nodes from AGORA genome-scale metabolic model; blue nodes from Exposome-Explorer.

The 251 predicted metabolites reported in the Exposome-Explorer database represent less than 11% of all the metabolites found in the gut bacteria GSMMs (Fig. 4). Remaining metabolites might be microbial metabolites that are also commonly produced in human cells/tissues and this would explain why they were not recognized as microbial metabolites in our literature search. In order to find microbial metabolites unique to the microbiome, the in silico predicted microbial metabolites were also compared to human metabolites in the Recon3D human GSMM reconstruction^23^. About 74% of the predicted gut microbial metabolites (*n* = 1727) were unique microbial metabolites not found in the human GSMM. These predicted metabolites cover a wide spectrum of the chemical space, just as microbial metabolites observed experimentally and documented in Exposome-Explorer (Fig. 5). The majority of predicted unique microbial metabolites (*n* = 1543) are not found in the Exposome-Explorer database. These metabolites are listed in Table S1 (Additional file 2) and could be further studied in new experimental studies.

## Discussion

Many metabolites are formed by the gut microbiota, either de novo or by biotransformation of endogenous and exogenous substrates such as dietary fibres or proteins. Some microbial metabolites such as short chain fatty acids and secondary bile acids have been widely studied and their role in disease risk is largely documented^3^. On the other hand, the role of many other microbial metabolites is still ignored. The purpose of the present work was to make an inventory of known microbial metabolites that can be found in humans. Once identified, the study of variations of their concentrations in populations may help understanding the role of the gut microbiota in health and diseases. The choice of biospecimens where they should be measured is another important issue. Most often, microbial metabolites have been measured in faecal samples. However, such samples are not easily collected in large cohort studies. Many microbial metabolites are absorbed through the gut barrier^10^ and can be measured in blood and urine where they could be used as surrogates of gut microbiota functionalities. To achieve that, it is also important to identify in the blood/urine metabolome, those metabolites that are of microbial origin.

## A first database on the microbial exposome

For the first time a list of manually curated gut microbial metabolites, composed of 462 compounds, is made publicly available in the Exposome-Explorer database. Three types of evidence of the microbial origin of metabolites were collected: (i) in vitro experiments with faecal samples showing that a particular metabolite can be produced by gut bacteria; (ii) in vivo manipulation of the gut microbiota with antibiotics showing a decrease of microbial metabolite concentrations; (iii) comparison of microbial metabolite concentrations in germ-free and conventional animals. The second and third types of evidence are particularly important because many microbial metabolites are also produced in human tissues and the proportion coming from the microbiota is most often unknown. A change of metabolite concentrations upon manipulation of the gut microbiota suggests a significant contribution of the microbiota to their production. Some metabolites like colibactin, a genotoxin known for its oncogenic role in colon cancer pathogenesis, or equol, a metabolite of soy daidzein with estrogenic properties, are known as metabolites exclusively formed by bacteria^18,24^. However, in a recent comparison of germ-free and conventionalized mice through metabolomics, only 5 of the 61 metabolites showing a reduction of concentrations in three different tissues in germ-free animals were absent in conventionalized mice^9^. This suggests that most metabolites measured in this particular study are not exclusive bacterial metabolites, but can also be formed in the animal tissues, or originate from the diet.

In Exposome-Explorer, a total of 108 metabolites simultaneously met an in vitro (production by gut bacteria) and an in vivo (reduction of concentrations by antibiotics or in germ-free animals) evidence supporting a microbial origin. These metabolites are therefore expected to be largely or exclusively produced by the gut microbiota (Table 2). Those microbial metabolites belong to diverse chemical classes with amino acids, phenylpropanoids, fatty acids, bile acids, phenols and indoles being most represented. This list is certainly not exhaustive, and more metabolites will be added in the future as more pieces of evidence are published.

For many of the remaining metabolites (*n* = 154), evidence on microbial origin is limited to in vitro studies with faecal samples. In vitro studies show that these metabolites can be formed by the microbiota but not necessarily that they are formed in vivo. For instance, the “Steroids and steroid derivatives” class includes 29 records of evidence on microbial origin which are for most of them derived from in vitro studies. In vivo evidence with antibiotics and germ-free animals is largely missing. Evidence on microbial origin based on in vitro studies with faecal samples also has some limitations. It only includes metabolites formed by the colon or caecal microflora, although some microbial metabolites can also be formed in the upper part of the gastrointestinal tract^25^. Some colonic microbial strains may not grow in in vitro experiments^26^ and some metabolites formed in vivo by these strains would be missed. In addition, these in vitro experiments cannot mimic the further transformation of microbial metabolites in the human tissues (phase 1 and 2 metabolism).

Evidence from manipulation of the microbiota with antibiotics or in germ-free animals also has its limitations. The absence of microbiota in germ-free animals may alter the metabolism of the host. Some metabolites show increase in their concentrations in germ-free animals^9^. They could either be precursors of microbial metabolites or indicators of a metabolic response to the absence of microbiota. Similarly, a reduction of the concentration of a metabolite cannot be seen as a definitive proof of its microbial origin, as it could also be explained by a metabolic response of the host. Therefore complementing this piece of evidence with in vitro experiments is important. Experiments with antibiotics may present similar limitations and care should be paid in such interpretations. As an example, antibiotics may inhibit liver canalicular bile salt export pump (Bsep) and this may result in a counterintuitive increase of concentrations of some secondary bile acids in plasma upon antibiotic treatment ^27^.

Another limitation of studies on manipulation of the microbiota is that they are most often conducted on murine models, which show differences in their gastrointestinal tract, might have a different microbiota and may differ in their metabolism when compared with humans. For instance, some bile acids are specific to mice and are largely absent in humans ^28^. It is thus important to check that these compounds have also been described in humans. We systematically searched for concentrations of the 457 microbial metabolites in human biospecimens: 2136 concentration values for 298 microbial metabolites can be found in the Exposome-Explorer database.

## The chemical space of the gut microbial exposome

The chemical space of the gut microbial exposome beyond metabolites unravelled in our literature search was further explored with an in silico approach. We compared GSMMs of humans and gut bacteria to identify metabolites that may be unique to the microbiota and not formed in humans. From a list of 2325 predicted gut microbial metabolites, 1543 were not present in Exposome-Explorer nor in the human GSMM (Additional File 2: Table S1). The number of predicted unique microbial metabolites could even be greater if we would take into account the metabolism of microbial metabolites by the host. For example, *p*-cresol, a microbial metabolite formed from tyrosine or hydroxyphenylacetate in the gut, is conjugated to its sulfate ester or glucuronide in the liver. These *p*-cresol metabolites are the forms detected in urine and plasma in human and animal experiments, but they are absent from the bacteria GSMM models. Furthermore, the list of predicted unique microbial metabolites inferred using the GSMMs is very likely underestimated as GSMMs are well known for containing gaps in the metabolites compared to the real metabolome^29^.

An overlap of 251 compounds was found between predicted microbial metabolites and the microbial metabolites curated in the Exposome-Explorer database. However, many predicted microbial metabolites (*n* = 1543) are not found in Exposome-Explorer. These include many secondary metabolites such as polyketides, terpenoids, and flavonoids, either formed de novo by the bacteria or formed from plant compounds, consumed as part of the diet and biotransformed by the microbiota. Secondary plant/microbial metabolites are characterized by considerable variations in their chemical structures. Many of them were used as substrates in experiments described in Exposome-Explorer (e.g. vitexin, rutin, quercetin, daidzein, ( +)-catechin, rosmarinic acid, thymol, resveratrol, deoxycholic acid), and more microbial metabolites will be found as more substrates are tested^9^.

## Gut microbial metabolites as a readout of gut microbiota functionalities

Compared to genes, proteins or transcripts, metabolites are the most downstream expression of phenotypes. As such, many gut microbial metabolites found in blood or in urine may constitute a useful read-out of microbiota functionalities in cohort studies. Many gut microbial metabolites are absorbed through the gut barrier and they can be found in blood where data on their concentrations have been collected. Some of them have been associated with disease outcomes in cohort studies, such as equol, a microbial metabolite of daidzein, associated with risk of colon cancer^30^, trimethylamine oxide associated with risk of rectal cancer^31^, or enterolactone, a microbial metabolite of dietary fibres, associated with endometrial cancer^32^.

Many authors have analysed microbial metabolites in faecal samples as a readout of the gut microbiota functionalities^2^. However, faecal samples are not easily collected in epidemiological studies and most often not available in large cohort studies. Measurements of bile acids and short chain fatty acids were compared in serum and faeces and poor correlations were observed between measurements in the two matrices^33^. The most likely explanation of this absence of correlations is the large heterogeneity of faecal samples^34^ coupled to the small amount of sample collected (200 mg) and analysed in the study. This heterogeneity also explains the large temporal variability when measuring bile acids and short chain fatty acids in stool repeat samples collected six months apart^33^. A much higher reproducibility was systematically observed when the same microbial metabolites were measured in serum repeat samples^33^, most likely explained by homogenization of microbial metabolites in several litres of blood. For these reasons, we would recommend measuring the microbial exposome in blood rather than in stool samples.

## Conclusions

This first database on the gut microbial exposome developed in this work can be used to identify microbial metabolites in metabolomics datasets or to develop specific assays to quantify them in clinical or epidemiological studies. This new release of the Exposome-Explorer database contains useful information to prioritize metabolites to be annotated or included in an assay, such as level of evidence on their gut microbial origin, biospecimens where they have been measured, analytical methods, concentrations, or reproducibility over time. The database also contains structural information such as InChiKey or SMILES that can be directly used for in silico mass spectrometry fragmentation and metabolite identification in metabolomics studies^35,36^. This kind of high-quality curated database is also very useful to train natural language processing and machine learning models, for example to automatically find microbiome-related metabolites in publications^37^. Finally, the list of in silico predicted unique microbial metabolites may guide researchers in the collection of new experimental evidence on their presence in humans. This new resource on the microbial exposome is focused on microbial metabolites. Other databases such as the Bacterial Isolate Genome Sequence Database (BIGSDB) and the KEGG Orthology database provide complementary data on nucleotide sequences in microorganisms colonizing humans and on corresponding molecular functions^38,39^. It will be important to link these different resources in the future to facilitate the integrative analysis of metabolomics and metagenomics data and to improve our understanding of the role of the gut microbiota in human health.

## Methods

### Data collection

Microbial metabolites and experimental evidence on their microbial origin were derived from manual collection of data in the scientific literature. Three types of evidence were collected.

- Produced by human faecal bacteria. Metabolites are produced during anaerobic or aerobic fermentation of dietary and non-dietary substrates by faecal samples, isolated individual faecal bacterial species or mixtures of faecal bacteria isolated from faecal samples collected in healthy individuals. Metabolites are measured in the culture medium. Nature of substrates, when added to the culture medium, was recorded, as well as names of bacterial species.
- Reduced by antibiotic treatment. Antibiotics are used to reduce the number of gut bacteria in humans or experimental animals. They can target specific types of bacteria or have a large spectrum of action. All antibiotics in these experiments, were administered orally. Concentrations are compared either before and after antibiotic treatment, or between antibiotic-treated and non-treated groups. These metabolites are measured in blood, urine, or faeces. We included metabolites which were reduced upon antibiotic treatment in healthy humans, monkeys, rats and mice. The nature of antibiotics was recorded. If specific substrates were tested, their names were provided.
- Reduced in germ-free animals. Germ-free mice and rats are compared with conventional animals. Germ-free animals may be conventionalized by having contact with human faeces, specific pathogen-free or conventional mice/rat faeces. We included metabolites which were reduced in blood, urine, faeces or in the content of intestine-linked organs of germ-free animals. The source of bacteria was recorded in the database. If specific substrates were tested, their names were provided.

A literature search was conducted in PubMed for each major chemical class of microbial metabolites. These classes, defined from different publications and review papers, included amino acids, branched chain amino acids, bile acids, bilirubins, choline derivatives, indoles, organic acids, polyphenols, polyamines, short chain fatty acids, and vitamins. Several additional classes were further included if compounds from these classes were identified, particularly in metabolomics studies in which a large diversity of metabolites can be measured. Literature search combined names of chemicals or chemical classes, with keywords related to gut, microbiota, germ-free animals, antibiotics, and type of biospecimens. Biospecimens in human and experimental animals were limited to blood, urine, faeces and gut contents. Concentration values in human biological samples were also collected as a proof of the microbial metabolite being detected in humans.

Data was collected from peer-reviewed publications. Review papers were not included in the database but were used to identify additional relevant publications. Data was manually collected from full-text original publications and inserted in the Exposome-Explorer database using the annotation interface previously described^17^ and adapted for the new data on microbial metabolites.

## Database implementation

Exposome-Explorer is a web application developed in Ruby on Rails (https://rubyonrails.org/). The data is stored in a MySQL database (https://www.mysql.com/). Chemical structures are hosted on the Wishart lab’s MolDB structure server (https://moldb.wishartlab.com/). Chemical information (e.g. IUPAC name, formula, molecular weight) is automatically calculated from the structures. Based on their structure, compounds are automatically classified with the ClassyFire webserver (http://classyfire.wishartlab.com/) which relies on the ChemOnt chemical taxonomy^40^. The ChemOnt taxonomy was developed for large public databases such as HMDB to facilitate unambiguous classification of chemicals^41^. The Exposome-Explorer website (http://exposome-explorer.iarc.fr/) is responsive and compatible with different systems and screen sizes, including mobile devices.

Some modifications have been brought to the user interface in order to seamlessly integrate the new microbial metabolite data with existing data. Two pages displaying the new microbial metabolite data have been incorporated below the ‘Biomarker data’ menu. The first page, ‘Microbial metabolites’, provides the list of 457 metabolites with available information on their microbial origin (http://exposome-explorer.iarc.fr/microbial_metabolites). For each metabolite, the number of experimental evidence types from 1 to 3 is indicated. The number of publications relative to each type of evidence is provided in three additional columns. Additional chemical information (e.g. identifiers from other chemical databases, chemical formula, molecular weight, InChIKey, SMILES) can be displayed in the ‘Microbial metabolites’ page via the ‘Show/Hide columns’ button. A last column in the page indicates if the metabolite has been described in humans. Concentrations in various biospecimens can then be found in the specific page of the corresponding metabolite, reached through clicking on the name of the metabolite.

The second page, ‘Associations with microbiota’, provides the list of 1848 raw database records of experimental evidence collected on the microbial origin of the metabolites, together with their bibliographic reference (http://exposomeexplorer.iarc.fr/microbial_metabolite_identifications). Additional information on organism (bacteria, humans or experimental animals) used in the experiment, biospecimen where the biomarker was identified, and substrate is also indicated. The nature of the antibiotic and bacterial source appears in hidden columns displayed with the ‘Show/Hide columns’ button.

## Microbial metabolites predicted by genome-scale metabolic models

The enzymatic reactions inferred from the genome sequences from the GSMMs are used to predict human and microbial metabolites. Predicted human metabolites were extracted from the Recon3D GSMM for the human organism^23^. Predicted gut microbial metabolites were obtained from the GSMMs part of the AGORA^21^ and MAMBO^42^ models.

The lists of metabolite names extracted from the GSMMs were formatted so that metabolite names could be reliably matched across lists of metabolites. The name formats were changed based on a set of common rules: prefixes like “cis-” became “z-” and symbols like “α-” became “alpha-”. The lists of metabolites from the GSMMs were manually cleaned-up by removing duplicates. Metabolites with a modelling function for AGORA, MAMBO, or Recon3D that were not real compounds (e.g. RNA) were excluded.

The list of predicted gut microbial metabolites, predicted human metabolites, and Exposome-Explorer microbial metabolites were matched based on a hierarchical matching starting by InChIKey, then ChEBI, then PubChem, then HMDB, then BiGG IDs, then MetaNetX. The leftover metabolites that did not match any metabolites were then matched using fuzzy matching between metabolite names. The list of fuzzy matched metabolites was checked manually to make sure matching was correct and to remove mismatched metabolites.

The metabolites that were set in the GSMMs as substrate metabolites for the bacteria (e.g. from diet) were manually excluded from the final list. However, due to the bi-directionality of most of the metabolic reactions in the GSMMs, it was not possible to discriminate metabolites consumed from the ones produced by the microbiome in a systematic way, and there might be some metabolite substrates left in the final list.

## Chemical similarity networks

The chemical similarity networks were generated and visualized using Cytoscape (v. 3.9.1)^43^. The similarity networks were computed from SMILES with a 0.8 Tanimoto coefficient using the *chemViz2* Cystoscape app^44^. The network calculation and figure generation in Cytoscape were automated with the RCy3 R package^45,46^.

## Supplementary Information

Supplementary Information 1.

Supplementary Information 2.

## Data Availability

All data is available in the Exposome-Explorer database (http://exposome-explorer.iarc.fr/).

## Abbreviation

GSMM: Genome-scale metabolic model
