# Supplementary material for: The human microbial exposome: expanding the Exposome-Explorer database with gut microbial metabolites
Source: Sci Rep. 2023 Feb 2;13:1946. doi: 10.1038/s41598-022-26366-w (PMC9894932; doi:10.1038/s41598-022-26366-w)
Supplement: Supplementary file 2 — Supplementary Information 2. [file 41598_2022_26366_MOESM2_ESM.docx]

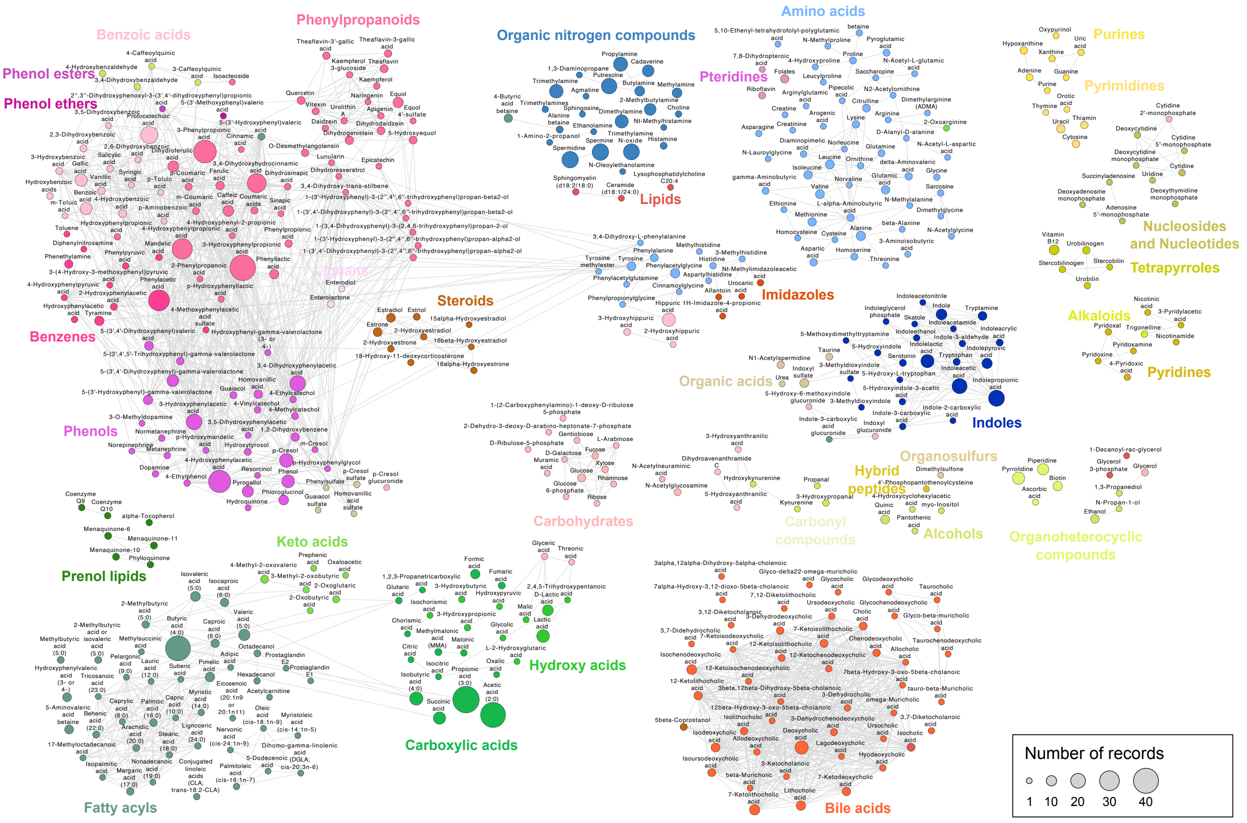

Fig. S1 Chemical similarity network of 457 microbial metabolites in the Exposome-Explorer database. Node size is proportional to the number of records supporting their microbial origin. The 33 upper-level ChemOnt classes appear with their own colour.

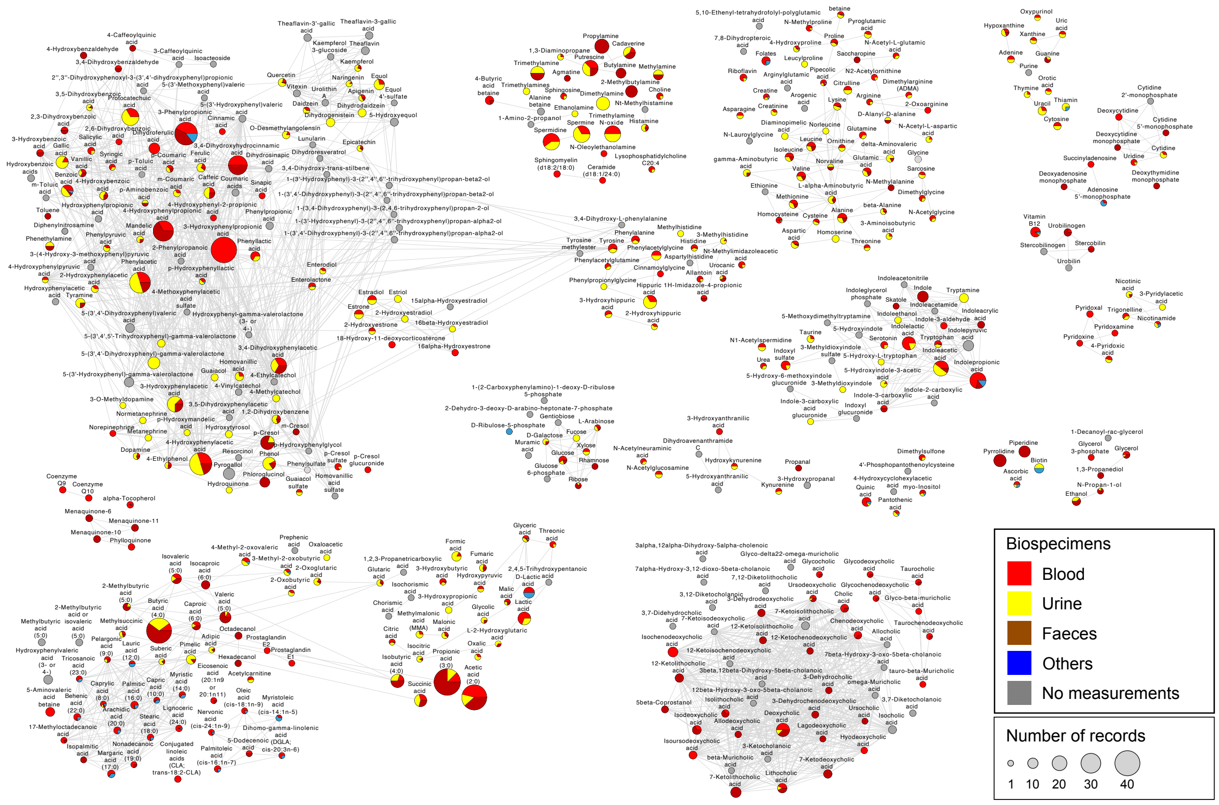

Fig. S2 Chemical similarity network of 457 microbial metabolites in the Exposome-Explorer database. Node size is proportional to the number of records supporting their microbial origin. Types of human biospecimens where metabolites have been measured are represented in red for blood, in yellow for urine, in brown for faeces, in blue for others types, and in grey when they were no measurements found in human.
